# P-1278. Risk Factors Associated with Post-Treatment Lyme Disease Syndrome: A Prospective Cohort Study on Long Island, NY

**DOI:** 10.1093/ofid/ofae631.1459

**Published:** 2025-01-29

**Authors:** David Kenison, Rudline G Zamor, Victoria A Bateman, Brigitte Maczaj, Sarath Nath, Bennadette Maramara, Michael D Lum, Dana Mordue, Luis A Marcos

**Affiliations:** Stony Brook University Hospital, Lake Grove, New York; Stony Brook University Hospital, Lake Grove, New York; Stony Brook University, Stony Brook, New York; Stony Brook Medicine, Stony Brook, New York; Stony Brook University Hospital, Lake Grove, New York; Stony Brook University Hospital, Lake Grove, New York; Stony Brook University Hospital, Lake Grove, New York; New York Medical College, Valhalla, New York; Renaissance School of Medicine at Stony Brook University, Stony Brook, New York

## Abstract

**Background:**

Lyme disease (LD) is caused by *Borrelia burgdorferi* transmitted by the Ixodes scapularis tick. Post-treatment Lyme disease syndrome (PTLDS) refers to a condition in which approximately 10-20% of optimally treated LD patients develop persistent symptoms of unknown pathophysiology. The epidemiological data on PTLDS is limited. This study aims to investigate the underlying risk factors associated with PTLDS.

**Table 1.**
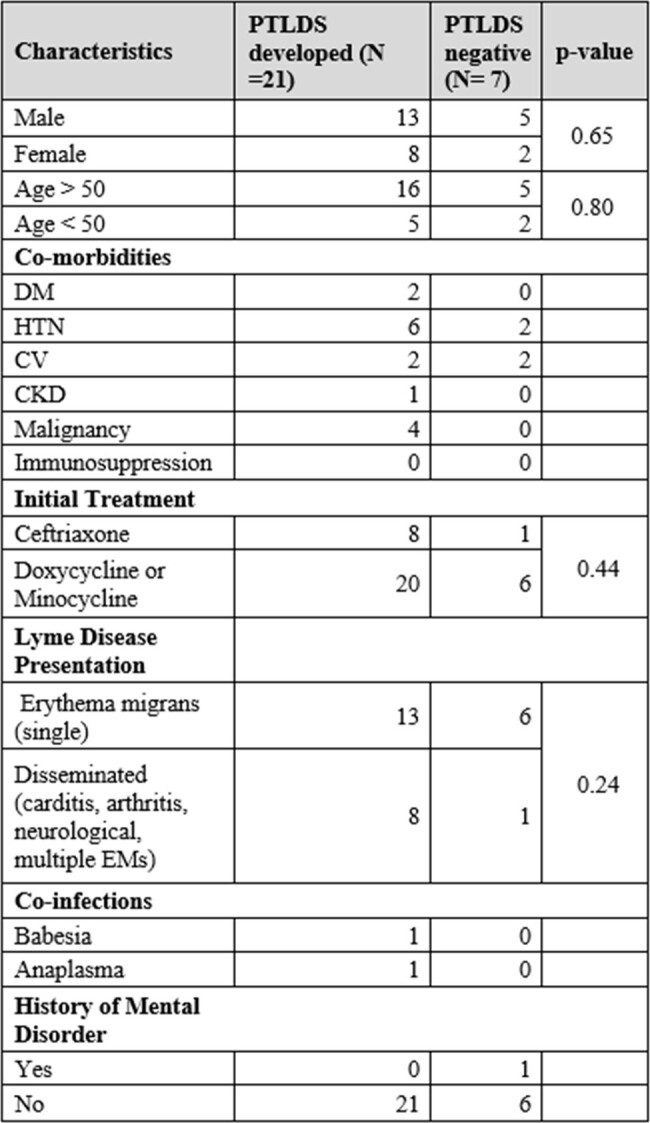
Patient characteristics in PTLDS developed and PTLDS negative groups and their associated p-values.

**Methods:**

This is an ongoing prospective cohort study of 47 adult patients with LD (CDC criteria) who were seen at Stony Brook Medicine in NY since 2021. Demographics, and pertinent epidemiological data, including immunocompromised status, coinfection, diagnosis, past medical history and treatment were collected. Twelve symptoms were assessed using the Visual Analogue Score (VAS) and Fatigue Severity Scores (FSS) at initial presentation and 1, 6- and 12-months post-treatment. PTLDS was defined based on at least one symptom present at 6 months. Continuous variables were described using means and standard deviations; categorical variables were described with frequencies and percentages.

**Table 2.**
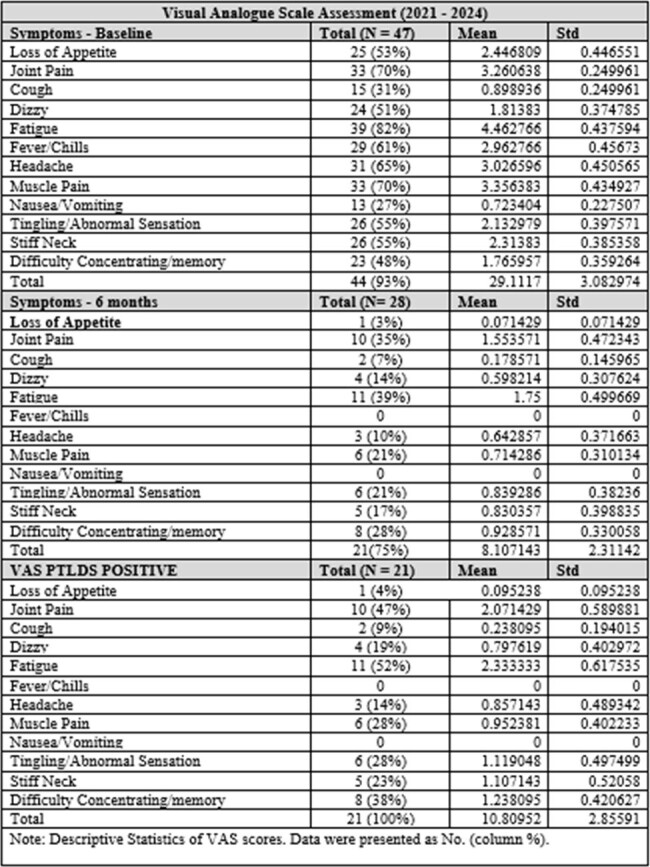
Symptoms reported by patients at 6 months and average Visual Analogue Scale (VAS) scores for each symptom.

**Results:**

Out of a total of 47 enrolled patients, 44 (female 31%) patients completed their initial VAS. At 6 months, fatigue had the highest frequency (39%). At the initial presentation, median total symptoms scores were 37 for disseminated Lyme (n=14) and 30.5 for erythema migrans (EM) (n=27). Compared to baseline, VAS total symptom scores decreased at 1 month for the disseminated (n=12, median= 6.5) and EM groups (n=25, median= 3.5). VAS total symptom score decreased at 6 months as well for the disseminated (n=10, median= 5.25) and EM groups (n=20, median=3.25). At each visit, the PTLDS group scored higher on the FSS compared to the No PTLDS group, indicating higher levels of fatigue. In both groups, FSS decreased over time.

**Table 3:**
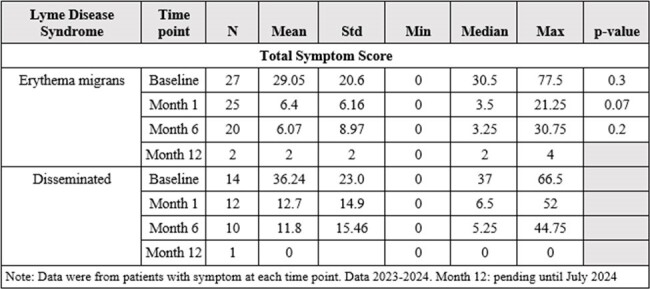
Total symptom score at each visit for erythema migrans versus disseminated. P-value calculated comparing LD syndrome disease at each corresponding time point.

**Conclusion:**

A trend of higher total symptom score was observed in disseminated patients compared to erythema migrans at 6 months post-treatment. Fatigue was the most common symptom reported and the PTLDS group consistently reported higher fatigue severity compared to the No PTLDS group.

**Table 4:**
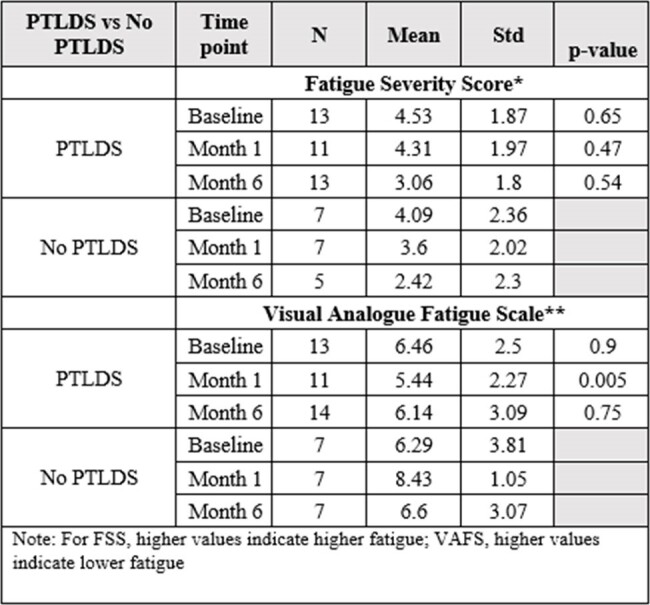
Fatigue Severity Score (FSS) and Visual Analogue Fatigue Scale (VAFS) at each visit for PTLDS and No PTLDS patients. P-value calculated comparing PTLDS and No PTLDS at each corresponding time point.

**Disclosures:**

**All Authors**: No reported disclosures

